# The enrichment of neutrophil extracellular traps impair the placentas of systemic lupus erythematosus through accumulating decidual NK cells

**DOI:** 10.1038/s41598-021-86390-0

**Published:** 2021-03-25

**Authors:** Meng Jiang, Nan Shen, Haibo Zhou, You Wang, Sihan Lin, Jiayue Wu, Wen Di

**Affiliations:** 1 Department of Obstetrics and Gynecology, Ren Ji Hospital, School of Medicine, Shanghai Jiao Tong University, Shanghai, 200127 China; 2 Shanghai Key Laboratory of Gynecologic Oncology, Shanghai, 200127 China; 3 Department of Rheumatology, Ren Ji Hospital, School of Medicine, Shanghai Jiao Tong University, Shanghai, 200127 China; 4 Shanghai Institute of Rheumatology, Shanghai, 200001 China; 5 State Key Laboratory of Oncogenes and Related Genes, Shanghai Cancer Institute, Ren Ji Hospital, School of Medicine, Shanghai Jiao Tong University, Shanghai, China

**Keywords:** Systemic lupus erythematosus, Reproductive disorders

## Abstract

Despite the advances made in the management of pregnancies in women with systemic lupus erythematosus (SLE), the rate of adverse pregnancy outcomes is still higher than that in the general population. In the last few years, neutrophil extracellular traps (NETs) were proven to be detrimental in both autoimmune diseases and placental injury. We investigated whether NETs could be detected in the placentas of pregnant individuals with SLE and explored the relationship between NETs and decidual natural killer cells (dNKs), which comprise the majority of immune cells at the maternal–fetal interface, using clinical samples and animal models. In this study, we found that the infiltration of NETs and dNKs, especially CD56^+^CD16^+^ NK cells, was significantly increased in pregnant individuals with SLE with placental insufficiency. In the murine models of SLE, the number of dNKs was significantly decreased due to the decreased formation of NETs affected by Ly6G. Moreover, the histopathological placental injury was reduced, with a remarkable increase in fetal birth weight. This study shows that NETs may contribute to immunological disorder in the placenta and the pathological changes in pregnancies with SLE, which provides a research basis for further explorations of the mechanism of SLE in placental impairment.

## Introduction

Systemic lupus erythematosus (SLE) is an autoimmune disease involving the production of autoantibodies and multiorgan pathological impairments. The peak age of onset is 15–40 years^1^, and nearly 90% of female patients are of reproductive age^2^. In recent decades, the diagnostic and therapeutic strategies for SLE have greatly improved; however, pregnancy complicated by SLE is still a high-risk pregnancy due to frequent complications, such as fetal loss, pregnancy-induced hypertension (PIH), preterm birth and intrauterine growth restriction (IUGR). The placentas of SLE pregnancies often present a histologic basis for inadequate blood flow, which could lead to damage to the placental vascular endothelium and placental insufficiency, consequently leading to adverse pregnancy outcomes (APOs)^3,4^. The success of a pregnancy relies on the regulation of the maternal–fetal immune system. An immune disorder at the maternal–fetal interface is considered to be one of the mechanisms of placental dysfunction resulting in APOs^5–7^.

Decidual natural killer cells (dNKs) account for approximately 70% of the immune cells at the maternal–fetal interface, comprise the largest specific immune cell population and decrease with increasing gestational age^8^. dNK cells are distinct from the majority of peripheral blood NK cells (pNKs). Approximately 90% of pNKs have a CD56^dim^CD16^+^ surface phenotype that represents cytotoxic NK cells^8^, but almost all dNKs are CD56^+^CD16^–^^9^, representing cytokine-producing NK cells. The presence of dNKs in early pregnancy can help the decidualization of the endometrium and the invasion of trophoblasts^9^. dNKs have been found in close proximity to the invading trophoblasts of the decidua and have been shown to produce vascular endothelial growth factor C (VEGFC), placental growth factor (PIGF), and angiopoietin 2 (ANG2). The increased number and dynamic phenotypical changes in dNKs help to initiate the delivery mechanism and may also induce various complications during pregnancy^10–12^, but the mechanism remains unclear. Wilczynski et al*.* reported that CD56^+^ NK cells showed an altered phenotype with a shift from the CD56^+^CD16^−^ phenotype to the more cytotoxic CD56^+^CD16^+^ phenotype in decidua from pregnancies complicated by preeclampsia (PE) compared with control samples^13^. The development of SLE is attributed to disorder of the innate immune responses. Although the levels of circulating NK cells are significantly lower in patients with SLE^14^, the role of dNKs in SLE-APO pathogenesis has rarely been examined.

Neutrophil extracellular traps (NETs) were first proposed in 2004. Brinkmann et al*.* found that a network structure was formed when circulating neutrophils were activated by bacterial endotoxins and inflammatory cytokines such as IL-8 or drugs, mainly composed of neutrophil elastase, myeloperoxidase (MPO), cathepsin G and proteinase 3^15^. NETs have the ability to trap and kill microbial triggers, including bacteria, viruses and fungi, by mediating toxic effects to the circulation or adjacent tissues. Although NETs are probably key for microbial defense, they can also be involved in the pathogenesis of a series of diseases. The role of NETs in the pathogenesis of autoimmune diseases has received increasing attention^16–18^. In patients with SLE, both an impaired ability to degrade NETs and a predilection of neutrophils to undergo NET release (or NETosis) have been observed^19^. In SLE, persistence of NETs may lead to prolonged exposure in an immunostimulatory context, which may further exacerbate the autoimmune response and form a vicious cycle^20^. Villanueva et al*.* suggested that low-density granulocytes (LDGs) had an increased capacity to kill endothelial cells through NETosis and noted that affected skin and kidneys from lupus patients were infiltrated by NETs^21^. NETs may therefore have a prominent role in inducing and perpetuating the vasculopathy observed in placentas from SLE cases, as well as fetal loss, IUGR and PE^22–27^. The risk of placental insufficiency in SLE patients is higher, and it may be closely related to NET-induced placental impairment. In addition, NETs have been shown to intimately shape the adaptive immune response at various levels, including the control of NK cell homeostasis^28^. However, it is still unknown whether NETs can cause placental pathology in SLE pregnancy by regulating dNKs.

MRL/lpr mice were proposed as a single gene SLE model in 1977^29^. MRL/lpr mice spontaneously develop a systemic, CD4^+^ T cell and macrophage dependent autoimmune disease and their histopathological and clinical features were similar to human SLE, including production of autoantibody and deposition of immune complexes in vital organs^30^. The anti-granulocyte receptor-1mAb, RB6-8C5, binds to Ly6G, which is present on neutrophils, has been widely used to deplete neutrophils in mice and to study the role of these cells in immune response^31^. Anti-Ly6G antibody (IA8) can be used for neutrophil depletion in vivo, which may affect the formation of NETs^32^.

We hypothesized that lupus pregnancies and their APOs would be associated with inflammatory histological features, as well as the infiltration of NETs and dNKs. Therefore, in this study, we characterized the distribution of NETs and dNKs in SLE pregnancies and determined the correlation between them in placental impairment.

## Methods

### Human samples

The placental tissues were taken from normal and pregnant women with SLE who voluntarily donated specimens to the biobank from August 2018 to April 2019. Approval was obtained from the ethics committee at Ren Ji Hospital, Shanghai Jiao Tong University School of Medicine, Shanghai, China. The exclusion criteria were smoking, fetal chromosomal abnormality, multiple pregnancy, severe infection before delivery, time of rupture to time of delivery greater than 24 h, chronic diseases (such as tumor, heart disease, hypertension or diabetes), and infectious diseases. All participants provided written informed consent and delivered by cesarean section. The placental collections were finished within 30 min of delivery.

APOs included one or more of the following^33^: (1) fetal loss or neonatal death unexplained by chromosomal abnormalities, anatomic malformation, or congenital infection; (2) preterm delivery or termination of pregnancy at less than 37 weeks due to placental insufficiency; (3) small-for-gestational-age (SGA) neonate, defined as one with a birthweight below the 10th percentile without anatomical or chromosomal abnormalities; and (4) PIH, namely, gestational hypertension (GH) or PE, diagnosed by the American College of Obstetricians and Gynecologists criteria^34^.

### Animal treatment and tissue collection

This study is in compliance with the ARRIVE guidelines for in-vivo studies carried out on animals. Pregnant MRL/lpr and C57BL/6 J mice, aged 8–10 weeks, were purchased from Shanghai Model Organisms Center, Inc. The case and wild-type (WT) mice were randomly divided into a test group and a control group on the day after the vaginal plug was observed. The mice in the test group received an intraperitoneal injection of 200 µg anti-mouse Ly6G (clone 1A8; BioXcell, West Lebanon, USA) once every 4 days, for a total of 4 injections. The control group was injected with an equivalent volume of 0.9% normal saline (NS) following the same protocol as that of the anti-mouse Ly6G injection. There were 4 mice in WT-Ly6G group, 6 mice in MRL/lpr-Ly6G group, 5 mice in WT-NS group and 6 mice in MRL/lpr-NS group. Mice were weighed every day. On days 16–18, their placentas were collected, and the weights of the fetal mice were measured. To accurately compare the birth weights of the different groups, the fetal body weights were measured using the following equation:

1 $$ {\text{The fetal body weight ratio}} = \frac{{{\text{fetal birth weight}}}}{{{\text{maternal weight}}\;{ }\left( {{\text{on the day vaginal plug was observed}}} \right)}} $$

Mice that died or had early pregnancy failure were excluded from the analysis.

### Histological review and immunohistochemistry

For determination of the anatomical features of the tissues at the maternal–fetal interface from murine and human placentas, sections were stained with hematoxylin and eosin (H&E). Placental apoptosis in mice was analyzed based on changes in nuclear morphology. Hematoxylin and eosin-stained slides and immunohistochemical stains were reviewed by 2 pathologists with expertise in perinatal pathology, with confirmation of diagnoses. In all cases, a 4-µm section was evaluated from both representative normal placental tissue and lesional placental tissue^35^.

Immunohistochemistry (IHC) was used for localization of MPO within both murine and human placentas. To this end, the 4 µm-thick sections were deparaffinized in xylene and washed in an ethanol concentration-gradient series. Then, the sections were rehydrated in phosphate-buffered saline (PBS). Antigen retrieval in tissues was performed using EDTA (pH 9.0) (Sigma, St. Louis, MO, USA) for 15 min at 90 °C, and sections were then blocked using 10% rabbit serum (Gibco, Grand Island, NY, USA) for 30 min at 37 °C. Afterward, slides were incubated overnight at 4 °C with anti-rabbit MPO antibody (Abcam, Cambridge MA, USA; 1:1000 dilution), rinsed three times with PBS, and incubated with goat anti-rabbit IgG-HRP (Abcam, Cambridge MA, USA; 1:200 dilution) for 50 min at 37 °C. After further washing with PBS, slides were stained with diaminobenzidine (DAB; Dako, Glostrup, Denmark) at room temperature for 10 min, and staining was stopped with distilled water. Sections were counterstained with hematoxylin for 5 min, dehydrated in alcohol and xylene and covered with neutral balsam^36,37^.

### Immunofluorescence staining

Immunofluorescence staining was used for localization of dNKs within human placentas. Placental tissues were isolated from pregnant women, immediately fixed in 4% paraformaldehyde for 4 h and washed in PBS. OCT-embedded frozen samples were cut into 4-μm sections and fixed with acetone (10 min). After washing three times with PBS, slides were blocked with 3% bovine serum albumin (BSA). Sections were then incubated with anti-rabbit MPO antibody (Abcam, Cambridge MA, USA; 1:600 dilution), anti-rabbit CD16 antibody and anti-mouse CD56 antibody (as above) overnight at 4 °C. Samples were then labeled with a fluorescein-conjugated secondary antibody (Abcam, Cambridge MA, USA) (dark, 45 min, 37 °C) and observed using a fluorescence microscope (Olympus, Tokyo, Japan). Tissue sections were examined at low and high magnification by randomly choosing 4 photomicrographs^38^.

### Flow cytometry

Single-cell suspensions were isolated by parenzyme digestion from murine placentas. For staining, single-cell suspensions were blocked with anti-FcR (clone 2.4G2, Bio X cell) and stained with antibodies for 30 min at 4 °C for cell surface staining. The following murine monoclonal antibodies were used: CD3-APC-Cy7 (clone 17A2), CD45.2-BV711 (clone 104), and NK1.1-PerCP-Cy5.5 (clone PK136). All antibodies were purchased from eBioscience (eBioscience, San Diego, CA, USA). Cells were washed twice with FACS buffer and subjected to flow cytometry. Samples were acquired on an LSRII flow cytometer using FACSDiva software (BD Biosciences, USA)^39^.

### Neutrophils and NETs

Neutrophils and NETs were identified by MPO immunohistochemistry. Placental sections were evaluated at 200×, with neutrophil counts obtained in 6 consecutive fields in which the maternal intervillous space was identified. An intact neutrophil was defined as MPO staining that was confined within the cytoplasm of the cell with a multilobulated nucleus was present. NETs were defined as detection of extracellular MPO detected in the presence of a disrupted, decondensed nucleus^26^. Intact neutrophils were counted, and NETs were analyzed by Image Pro Plus software and quantified with integrated optic density (IOD). Because NETs were activated from neutrophils, NETosis was assayed as the ratio of NETs (IOD)/intact neutrophils (n).

### Statistical analysis

The immunofluorescence in human placentas was analyzed with Image Pro Plus software (Media Cybernetics, Inc., Rockville, USA). Flow cytometric analysis of murine placentas was performed with FlowJo Version 10.1 software (FlowJo, LLC. BD, USA). Continuous variables are presented as the mean ± standard errors of the mean (SEM), and statistical comparisons between groups were performed using chi-square tests, Mann–Whitney U tests or Kruskal–Wallis H tests. Spearman’s tests were used for correlation studies. All statistical analyses were carried out using the SPSS v25.0 software program (SPSS, Inc., Chicago, USA). A value of *P* < 0.05 was considered significant.

### Ethics statement

All aforementioned procedures were in accordance with the Helsinki Declaration of 1975, as revised in 2008 (5), concerning human and animal rights and adhered to the policy concerning informed consent as shown on Springer.com. The institutional review board of Renji Hospital, School of Medicine, Shanghai Jiao Tong University approved the use of available human placentas (KY2019-138) and animal experiments (A-2018–022). Informed consent was obtained from all patients for inclusion in the study. Each case has been anonymized in this report. All institutional and national guidelines for the care and use of laboratory animals were followed.

## Results

### Population characteristics

Placentas from 19 healthy controls, all of whom were scheduled for planned delivery by cesarean section, were compared with placentas from 22 pregnancies complicated with SLE. The average age of all pregnant women was 30.3 ± 3.8 years (23–39 years), and the average gestational age was 268.5 ± 8.6 days (240–281 days). Compared with those of the control group, the average gestational age and fetal birth weight were decreased significantly in the SLE group.

There were 12 cases (54.5%) of APOs (SLE-APO group) and 10 cases (45.5%) without APOs (SLE-Neg group) among the SLE pregnancies. Detailed demographic and clinical data are presented in Table 1. More than one APO occurred in two patients (one complicated by SGA, PE and preterm delivery; the other complicated by SGA and preterm delivery). The two SLE groups had significantly shorter mean gestational ages and lower fetal birth weights than the control group (*P* < 0.01), but there were no statistically significant differences between the two SLE groups. Six individuals in the SLE group had lupus nephritis, but none of them had baseline renal impairment.

**Table 1:** Characteristics of the study population.

Characteristics	Control (n = 19)	SLE-Neg (n = 10)	SLE-APO (n = 12)	P value (for all)
**Maternal**				
Age (mean; SEM)	30.4 (3.4)	29.5 (3.9)	30.7 (4.7)	0.744
Lupus nephritis	n/a	2 (20.0%)	4 (33.3%)	/
**Fetal**				
Gestational age, days (mean; SEM)	273.3 (6.0)^▲^	266.3 (5.9)	262.7 (10.0)	< 0.01**
Weight, g (mean; SEM)	3327.9 (281.2)^▲^	3054.0 (259.3)	2644.2 (499.2)	< 0.01**
**APOs**				
Preterm delivery	NA	NA	2 (16.7%)	/
PIH	NA	NA	7 (58.3%)	/
SGA	NA	NA	6 (50.0%)	/
Fetal loss	NA	NA	NA	/

*SEM*  standard error of the mean, *PIH *pregnancy-induced hypertension, *SGA *small-for-gestational-age, *NA *not applicable.

^▲^Statistically different from the SLE-Neg group and the SLE-APO group.

**P < 0.01.

### Pregnancy outcomes of mice

The average age at conception in the case group of female mice was higher than that in the WT group (9.5 ± 0.7 weeks vs 8.4 ± 0.5 weeks, *P* < 0.01), indicating that MRL/lpr had delayed conception compared to that of the WT control. Consequently, the average weight of the case group at conception was higher than that of the WT group (27.3 ± 1.8 g vs 20.3 ± 1.4 g, *P* < 0.01). There were no significant differences in the survival rates of the fetuses among the four groups (WT-NS vs WT-Ly6G vs MRL/lpr-NS vs MRL/lpr-Ly6G; χ2 = 4.819, *P* > 0.05). The fetal body weight ratios were higher after Ly6G injection than after NS injection in the same strains (*P* < 0.05), but the highest ratio was in the WT-Ly6G group and the lowest was in the MRL/lpr-NS group. There was no significant difference in the body weight ratio between the WT-NS group and the MRL/lpr-Ly6G group.

### The placentas of patients with SLE and MRL/lpr mouse pregnancies show pathological impairment

**Figure 1:**
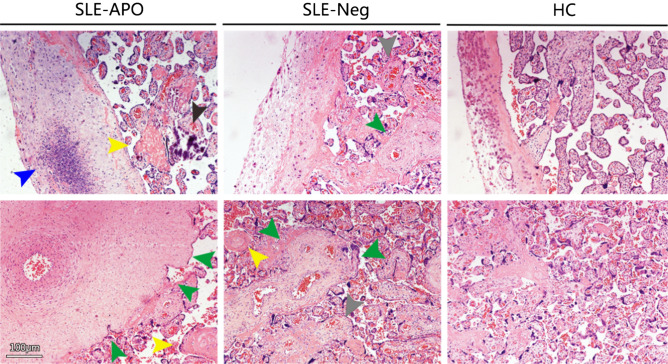
Representative images of human placenta HC: healthy control group. Scale bar = 100 μm. Vascular pathology at the maternal–fetal interface such as decidual vasculopathy (green arrows), villous infarction or fibrin deposition (yellow arrows) was observed in SLE groups. Many inflammatory cells infiltrated the placental bed in the SLE-APO group (blue arrow). Increased perivillous fibrin (grey arrows) consequently reduced the villous volume and surface area for maternal–fetal exchange. The black arrow refers to calcification.

As shown in Fig. 1, histologic evidence of malperfusion (increased syncytial knots, decelerated villous maturity, and villous agglutination), decidual vasculopathy, and intervillous hemorrhage were observed in all disease groups. The abovementioned abnormal placental pathologic findings were observed more often in the SLE-APO group than in the SLE-Neg group. The SLE-APO group also showed evidence of infarction and increased perivillous fibrin deposition. Inflammatory cell infiltration at the basal plate and enlarged villi space were observed, as well as immature and multivascular villi. The blood vessel wall was thickened in the placentas of the SLE-APO group, which might induce decreased uteroplacental blood perfusion and hypoxia–ischemia in the placenta.

A spectrum of histologic abnormalities was observed on microscopic review (Table 2); 63.6% of disease cases (14/22), including 5/10 cases in the SLE-Neg group (50.0%) and 9/12 cases in the SLE-APO group (75.0%), showed at least one abnormal placental pathologic finding (*P* < 0.01 for all).

**Table 2:** Histopathologic findings in human placenta.

Characteristics	Control (n = 19)	SLE-Neg (n = 10)	SLE-APO (n = 12)	P value (for all)
Decidual vasculopathy	1 (5.3%)	3 (30.0%)	4 (33.3%)	0.099
Infarction	0 (0.0%)	2 (20.0%)	4 (33.3%)^□^	0.033*
Intervillous thrombi	0 (0.0%)	1 (10.0%)	2 (16.7%)	0.207
Chronic villitis	1 (5.3%)	3 (30.0%)	7 (58.3%) ^□^	< 0.01**
Malperfusion	0 (0.0%)	1 (10.0%)	5 (41.7%)^□^	< 0.01**
Increased perivillous fibrin	1 (5.3%)	3 (30.0%)	5 (41.7%)^□^	0.045*

^□^Statistically different from the control group.

*P < 0.05; **P < 0.01.

**Figure 2:**
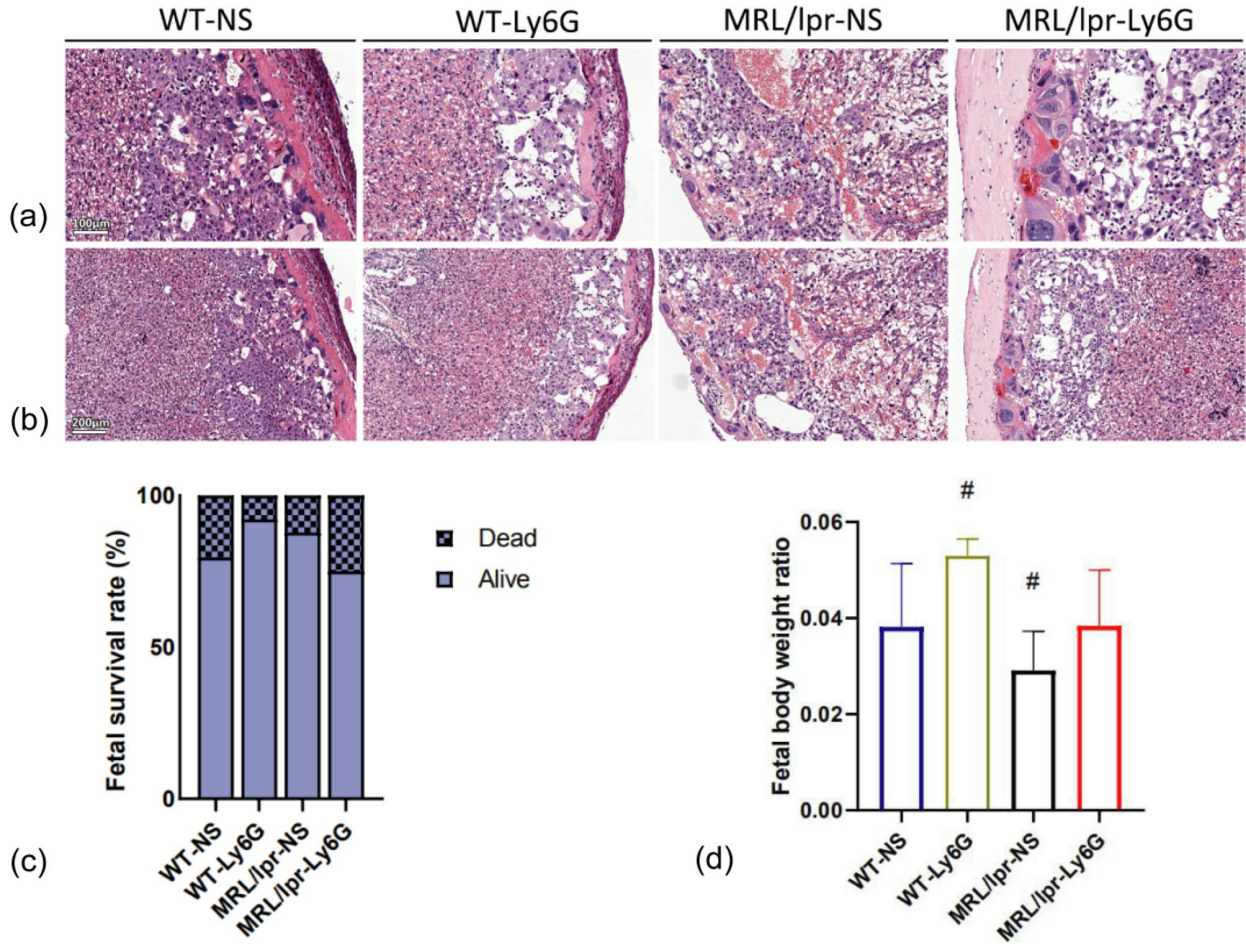
Representative images of the murine placenta WT-NS group (n = 5); WT-Ly6G group (n = 4); MRL/lpr-NS group (n = 6); MRL/lpr-Ly6G group (n = 6). (**a**) Scale bar = 100 μm. (**b**) Scale bar = 200 μm. (**c**) Fetal survival rate in different mouse groups. (**d**) Fetal body weight ratios in different mouse groups; Horizontal lines with bars show the mean ± SEM. ^#^*P* < 0.05 (compared with the remaining groups).

Similar manifestations were found in mouse placentas. However, unlike human placentas, mouse placentas are characterized by apoptotic or necrotic cells. There was no obvious pathological damage in the WT group after Ly6G injection, indicating that Ly6G rarely had toxic or side effects on the placenta. Additionally, the pathological damage in the placenta of the MRL/lpr-Ly6G group was reduced compared with that of the MRL/lpr-NS group (Fig. 2a,b), in accordance with higher fetal body weight ratios in the MRL/lpr-Ly6G group (Fig. 2d). There was no significant difference in the fetal survival rate among the groups (Fig. 2c).

### A higher level of NETosis in the intervillous space of SLE-APO patients and MRL/lpr mouse groups than in that of the controls

**Figure 3:**
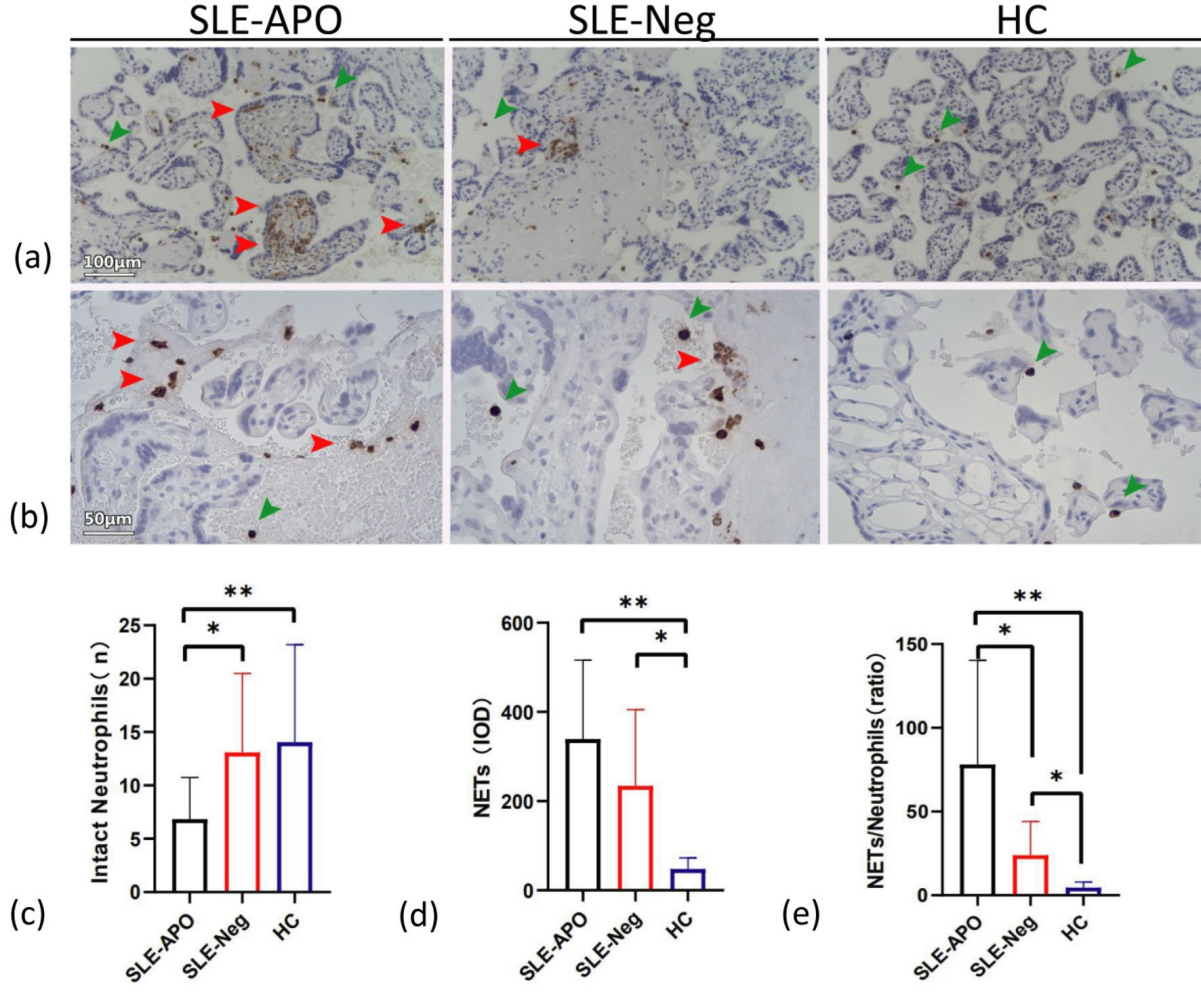
Comparison of MPO staining in human placentas. (**a, b**) Representative images of MPO staining. (**a**) Scale bar = 100 μm. (**b**) Scale bar = 50 μm. (**c**) Intact neutrophil quantification. (**d**) IOD of NETs. (**e**) The magnitude of the NET/intact neutrophil ratio. Horizontal lines with bars show the mean ± SEM; Intact neutrophils (green arrows); NETs (red arrows); HC: healthy control group; *P < 0.05, **P < 0.01.

To determine whether NETs could cause the placental impairment, we quantified the level of NETosis after immunohistochemistry staining of placental sections. Infiltration of NETs and neutrophils was observed in the placental intervillous space of the SLE groups compared with the control group (Fig. 3a, b). The intact neutrophil quantification in the SLE-APO group and IOD of NETs in the control group were lowest (Fig. 3c, d). Additionally, the magnitude of the NET/intact neutrophil ratio was highest in the SLE-APO group, and there were significant differences among the three groups (*P* < 0.05) (Fig. 3e), which indicated that large neutrophilic infiltrate in the intervillous space of SLE pregnancies, especially those complicated with APOs, might be a prominent feature that is not present in normal pregnancies.

**Figure 4:**
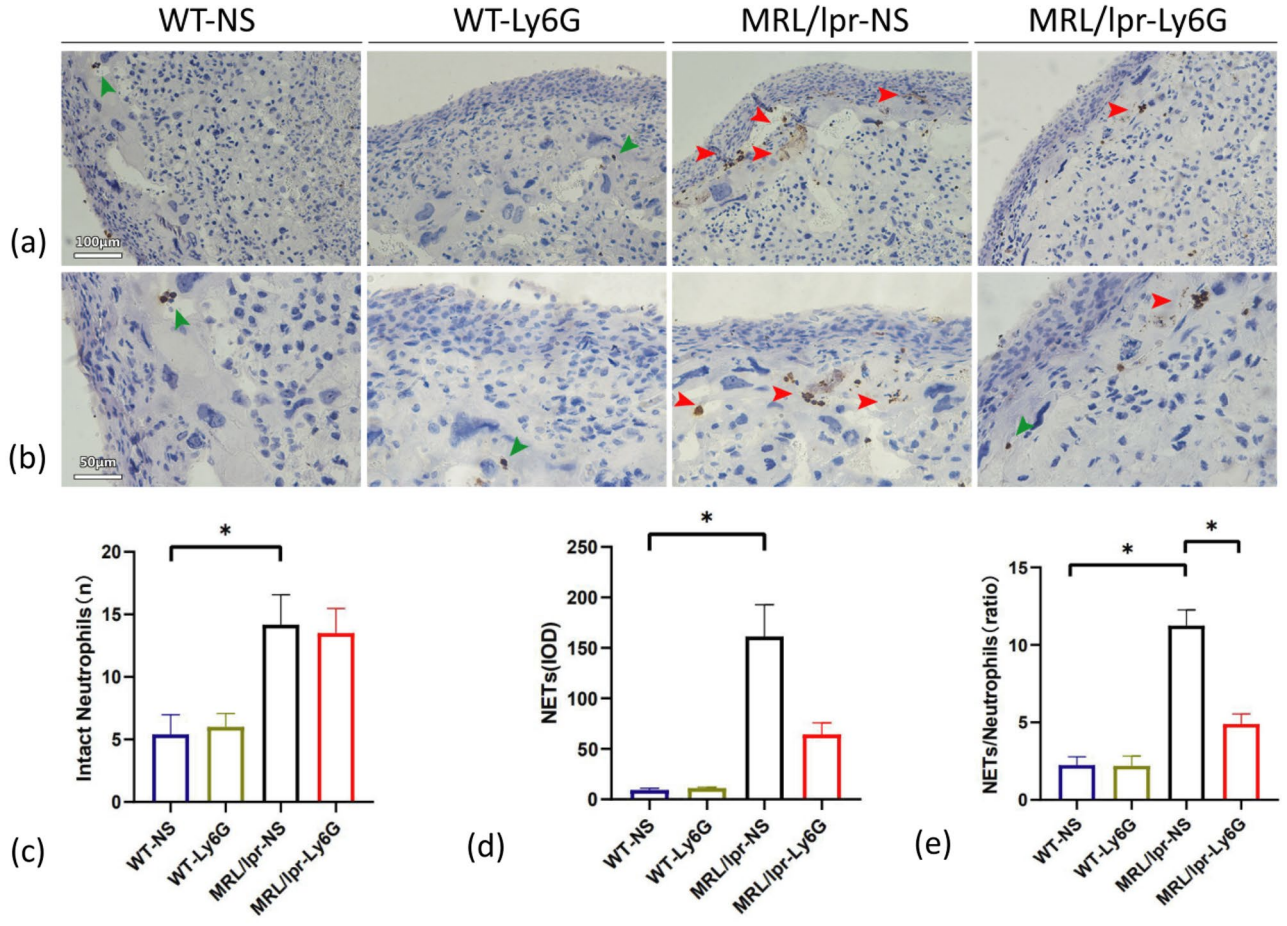
Comparison of MPO staining in murine placentas (**a**, **b**) Representative images of MPO staining. (**a**) Scale bar = 100 μm. (**b**) Scale bar = 50 μm. (**c**) Intact neutrophil quantification. (**d**) IOD of NETs. (**e**) The magnitude of the NET/intact neutrophil ratio. Horizontal lines with bars show the mean ± SEM; Intact neutrophils (green arrows); NETs (red arrows); *P < 0.05.

In the murine models, neutrophils were rarely observed in the placentas of the WT groups, similar to NETs (Fig. 4a, b). In addition, NETs and neutrophils in the MRL/lpr groups were significantly increased. NETs were reduced after Ly6G injection compared with NS injection in the MRL/lpr group, although there was no significant difference (*P* > 0.05) (Fig. 4c, d). The MRL/lpr-NS group had the highest NET/intact neutrophil ratio, with significant differences among the groups (*P* < 0.05) (Fig. 4e).

### Increased dNKs are associated with the infiltration of NETs in the intervillous space of the placenta

**Figure 5:**
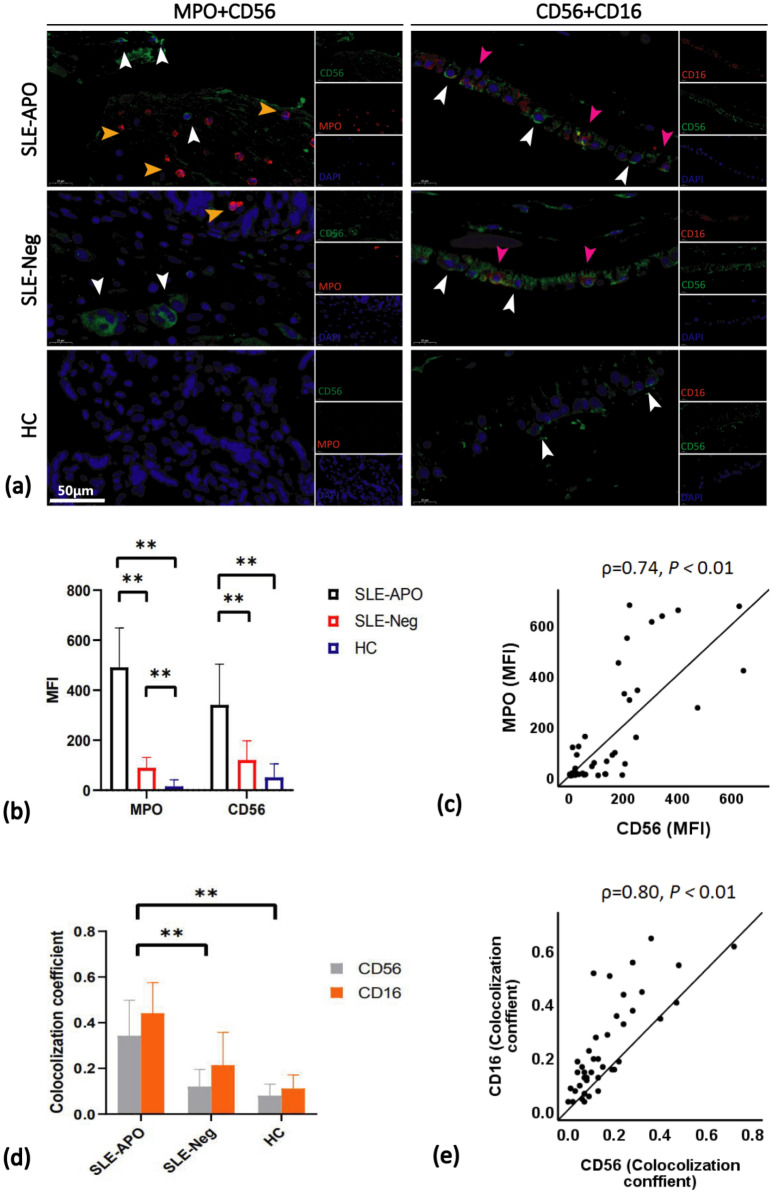
Increased MPO, CD56 and CD16 distribution in the placenta of SLE pregnancies. (**a**) Representative immunofluorescence staining demonstrating the distribution of NETs, CD56^+^ NK cells and CD56^+^CD16^+^ dNKs. (**b**) MFI of MPO and CD56 per field for each section. (**c**) Positive correlation between the MFI values of MPO and CD56. (**d**) The colocalization coefficients of CD56 and CD16 per field for each section. (**e**) Positive correlation between the colocalization coefficients of CD16 and CD56. Horizontal lines with bars show the mean ± SEM; NETs (orange arrows); CD56^+^ NK cells (white arrows); CD56^+^CD16^+^ dNKs (pink arrows). **P < 0.01. Scale bar = 50 μm.

Next, we determined whether the infiltration of NETs in the intervillous space of placentas might be associated with changes in immune cells such as dNKs, which are the largest specific immune cell population at the maternal–fetal interface. As we thought that the distribution of NETs in the placenta increased in the SLE-APO group, we compared the numbers of dNKs in control and SLE pregnancies. The relevance of NETs and CD56^+^CD16^+^ dNKs was confirmed by immunofluorescence analysis of the localization of MPO, CD56 and CD16 on serial placental sections (Fig. 5a). The median fluorescence intensity (MFI) of MPO was positively associated with that of CD56 (ρ = 0.74, *P* < 0.01, Fig. 5c), which was the highest in the SLE-APO group (Fig. 5b). Furthermore, the colocalization coefficients of CD56 and CD16 showed a positive correlation (ρ = 0.80, *P* < 0.01, Fig. 5e), and this parameter was the highest in the SLE-APO group (Fig. 5d). In view of the above findings, NETs might be positively correlated with CD56^+^CD16^+^ dNKs.

**Figure 6:**
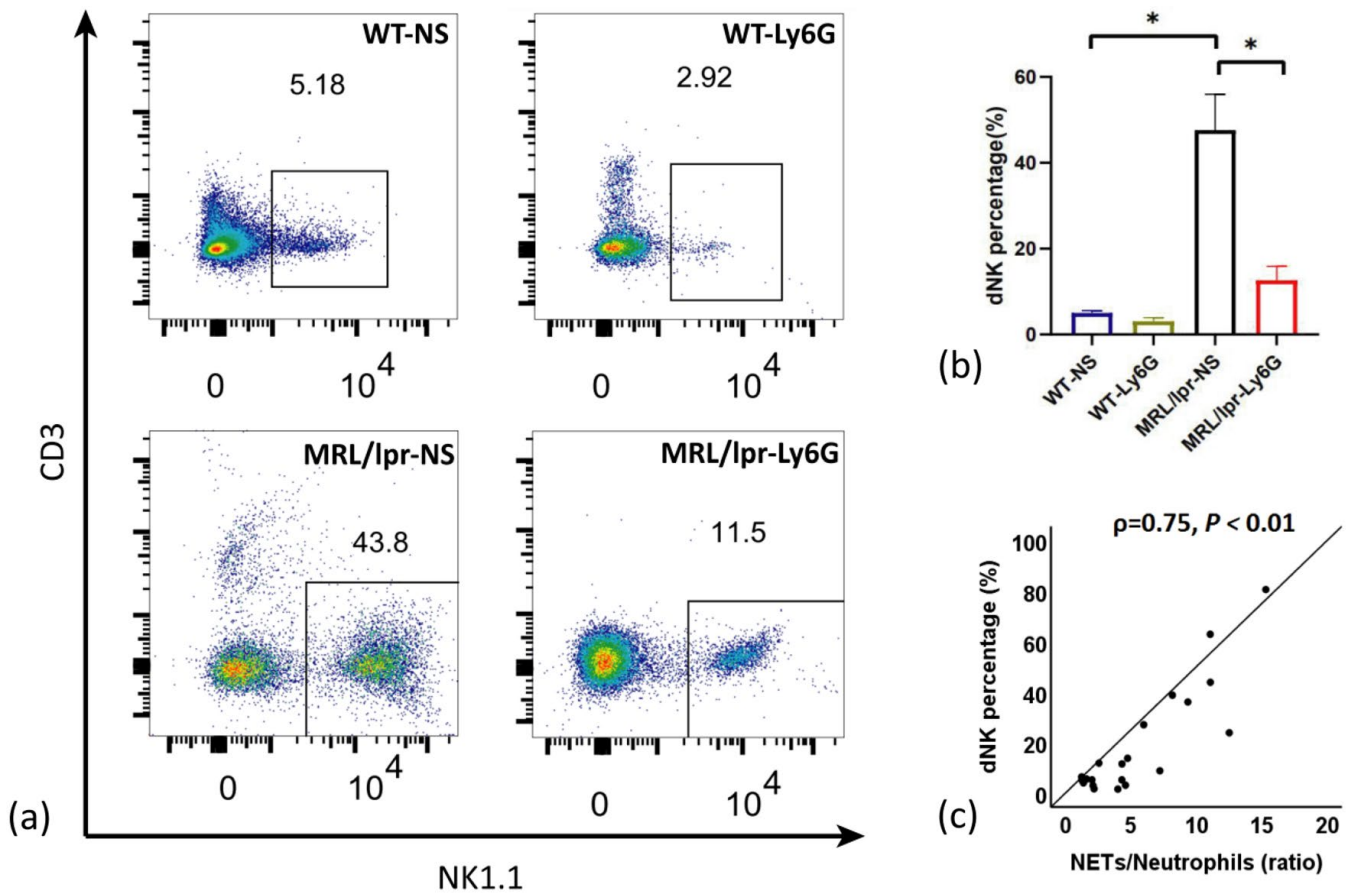
Comparison of dNK levels in pregnant mice of each group. (**a**) Representative plots of flow cytometry in each group. (**b**) Frequency of dNKs observed in each mouse group; (**c**) Positive correlation between the infiltration of NETs and dNK. Horizontal lines with bars show the mean ± SEM; *P < 0.05.

The dNKs in the placentas of the four mouse groups were compared by flow cytometry. As shown in Fig. 6, the dNK level of the MRL/lpr-NS group was significantly higher than those of the other three groups (*P* < 0.05, Fig. 6a, b). To investigate the association between NETs and dNKs, we used Spearman correlation analysis and identified a positive correlation between the infiltration of NETs and an increased dNK number (ρ = 0.75, *P* < 0.01, Fig. 6c). These results suggest that the numbers of dNKs are reduced when the course of NETosis is obstructed. Therefore, it is plausible that NETs may influence the level of dNKs during pregnancy, which is critical for adverse pregnancies. Furthermore, we propose that the infiltration of NETs observed in SLE pregnancies may be a factor underlying defective immune homeostasis that affects the level of dNKs at the maternal–fetal interface.

## Discussion

Characterization of NETs and dNKs in the placenta of SLE pregnancies may provide insights into the pathogenesis of APOs. In the present study, we found that the levels of NETs and dNKs were remarkably increased in the SLE placenta, especially in those with APOs. In an in vivo experiment of SLE murine models, dNK cells were significantly decreased due to the decreased formation of NETs affected by Ly6G, with a remarkable improvement in histopathological placental injury. These findings indicated that the infiltration of NETs at the maternal–fetal interface might contribute to placental impairment of SLE by cooperation with dNKs and cause corresponding APOs.

The placenta is a highly vascularized target organ during SLE pregnancy. Therefore, it is most likely to exhibit the earliest signs of vascular compromise leading to placental insufficiency. Placental insufficiency with resultant ischemia may manifest as not only PE but also IUGR with SGA infants and stillbirth. In this study, we found increased inflammatory infiltration at the maternal–fetal interface and villi space of the placenta in SLE pregnancies, accompanied by fibrin deposition and small infarcts, which was the same as in previous studies of SLE^3,19,40^. The results in the MRL/lpr murine placenta were consistent with those of humans and contributed to the lower birth weight of fetuses.

An early suggestion that NETs may play a role in pregnancy-related disorders was made in the context of PE^22^. However, SLE and PE share the same pathological placental mechanism, including vascular endothelial injury and inflammation. An increased level of NETosis was found in PE, probably activated by placentally derived syncytiotrophoblast microvillous membrane microparticles (STBM)^23^. In SLE, increased endothelial cell apoptosis is associated with a novel proinflammatory subset of lupus neutrophils, which are especially predisposed to NET formation^41^. These results suggested that NET infiltration may contribute to placental insufficiency in pregnancies complicated with SLE. Mader et al. revealed that significantly increased NETs were found infiltrating placental intervillous spaces in SLE and PE cases^26^. Additionally, NETs were associated with maternal vasculitis, laminar decidual necrosis, maternal–fetal interface hemorrhage and nonocclusive fetal thrombotic vasculopathy. Other studies suggested that NETs may be involved in recurrent fetal loss mediated by anti-phospholipid antibodies, which is associated with inflammatory activation via the complement system^42^. Furthermore, Bertin et al*.* suggested that NK aggregation was crucial for thrombus development by promoting the formation of NETs by neutrophils^43^.

dNKs reach their maximum number at the maternal–fetal interface during first pregnancy and then progressively decrease, resulting in small numbers at term^44^. The increase in dNK number and the dynamic phenotypical change in dNKs could cause complications during pregnancy, such as recurrent spontaneous abortion and PE, but the precise mechanism is not established. Previous researchers suggested that in the human endometrium and decidua, the prominent NK cells are CD56^bright^CD16^−45–48^, which may participate in trophoblast invasion and spiral artery remodeling during the first trimester^49^. However, studies investigating dNK cell numbers and functional shifts during pregnancy have shown varying results. Elevated numbers of CD56^+^ NK cells within the decidua were noted in some research on PE and IUGR^49–52^. Moreover, CD56^+^ CD16^+^ NK cells were proven to be predominant in the decidual specimens of the studied women with repeated unexplained miscarriage^12,37^. In contrast, other studies have found that decreased dNKs are crucial for failed placentation and insufficient blood supply to the developing fetus. It was suggested that CD56^+^ NK cells declined in placental bed biopsies from women with PE and IUGR^53,54^. The lack of clarity among various studies as to whether dNK cell numbers are reduced in APOs likely reflects differences in sampling, analysis, and disease severity^55^.

In this study, we showed that the dNK numbers, especially CD56^+^CD16^+^ NK cell numbers, were significantly increased in SLE pregnancies with SGA, PIH, or preterm birth related to placental insufficiency, of which poor perfusion and tissue damage had been found in the placenta. The number of dNKs should be in the downward trend during normal pregnancy, but it still maintains a high level in placental impairments. It has been indicated that the recruitment of dNK cells to the early decidua helps vascular remodeling and promotes successful pregnancy, while overstimulation by cytokines and the continuous secretion of dNKs in late pregnancy may have a damaging effect on the placenta. The above situation has also been verified in SLE animal models, while the infiltration of NETs in the placenta has been positively correlated with the dNK population. The combination of Ly6G and neutrophils may affect the formation of NETs accompanied by significantly decreased dNKs. Moreover, the histopathological placental injury is improved, with a remarkable increase in fetal murine birth weight. NETs appear to play a key role in regulating dNK subpopulations and quantity. However, we do not exclude the contribution of other cells at the maternal–fetal interface, including various immune and non-immune cells^56^. This study uses clinical samples and animal models to show that the joint action of NETs and dNKs may contribute to the pathological damage of the placenta in SLE pregnancy. Our results provide a mechanistic link between NETs and the increased risk of complications in SLE and a research basis for further exploring the impact of NETs on immune disorders of the placenta. However, these findings do not include a more in-depth understanding of the molecular mechanism.

In conclusion, our findings reveal the correlation between the presence of NETs and dNK cells in the pathogenesis of SLE-mediated placental impairment. These results improve our understanding and will guide future studies of targeted therapies for placental injury in SLE pregnancy. Further investigations are needed to specify the mechanism between NETs and dNKs in physiological and pathological pregnancy.

## Supplementary Information

Supplementary Information
