# Supplementary material for: The enrichment of neutrophil extracellular traps impair the placentas of systemic lupus erythematosus through accumulating decidual NK cells
Source: Sci Rep. 2021 Mar 25;11:6870. doi: 10.1038/s41598-021-86390-0 (PMC7994714; doi:10.1038/s41598-021-86390-0)
Supplement: Supplementary file 1 — Supplementary Information [file 41598_2021_86390_MOESM1_ESM.docx]

**The enrichment of neutrophil extracellular traps impair the placentas of systemic lupus erythematosus through accumulating decidual NK cells**

Meng Jiang^1,2^; Nan Shen^3,4^; Haibo Zhou^3,4^; You Wang^1,2^; Sihan Lin^1,2^; Jiayue Wu^1,2,^*^Δ^*; Wen Di ^1,2,5,^*^Δ^*

^1^Department of Obstetrics and Gynecology, Ren Ji Hospital, School of Medicine, Shanghai Jiao Tong University, Shanghai 200127, China. ^2^Shanghai Key Laboratory of Gynecologic Oncology, Shanghai 200127, China. ^3^Department of Rheumatology, Ren Ji Hospital, School of Medicine, Shanghai Jiao Tong University, Shanghai 200127, China. ^4^Shanghai Institute of Rheumatology, Shanghai 200001, China. ^5^State Key Laboratory of Oncogenes and Related Genes, Shanghai Cancer Institute, Ren Ji Hospital, School of Medicine, Shanghai Jiao Tong University;

^Δ^ **Correspondence to Jiayue Wu** Email: [janet_wu_jiayue@163.com](mailto:janet_wu_jiayue@163.com), and **Wen Di**, Email: [diwen163@163.com,](mailto:diwen163@163.com,)

**Supplementary Table S1. Abbreviations**

| SLE | systemic lupus erythematosus |
| --- | --- |
| APOs | adverse pregnancy outcomes |
| NETs | neutrophil extracellular traps |
| dNKs | decidual natural killer cells |
| PIH | pregnancy-induced hypertension |
| IUGR | intrauterine growth restriction |
| pNKs | peripheral blood NK cells |
| VEGFC | vascular endothelial growth factor C |
| PIGF | placental growth factor |
| ANG2 | angiopoietin 2 |
| PE | preeclampsia |
| MPO | myeloperoxidase |
| LDGs | low-density granulocytes |
| SGA | small-for-gestational-age |
| GH | gestational hypertension |
| WT | wild-type |
| NS | normal saline |
| H&E | hematoxylin and eosin |
| IHC | immunohistochemistry |
| PBS | phosphate-buffered saline |
| DAB | diaminobenzidine |
| BSA | bovine serum albumin |
| IOD | integrated optic density |
| SEM | standard errors of the mean |
| NA | not applicable |
| HC | healthy control group |
| STBM | syncytiotrophoblast microvillous membrane microparticles |

**Supplementary Table S2. Pregnancy outcomes of mice**

| Characteristics | WT-NS  (n=5) | WT-Ly6G (n=4) | MRL/lpr-NS  (n=6) | MRL/lpr-Ly6G  (n=6) | P value  (for all) |
| --- | --- | --- | --- | --- | --- |
| Maternal |  |  |  |  |  |
| Average age at conception, weeks (mean; SEM) | 9.5 (0.7) | | 8.4 (0.5) | | <0.01** |
| Average weight at conception, g (mean; SEM) | 27.3 (1.8) | | 20.3 (1.4) | | <0.01** |
| Fetal |  |  |  |  |  |
| Live/dead | 8/31 | 3/35 | 5/36 | 8/24 | 0.186 |
| Fetal body weight ratio (mean; SEM) | 0.038 (0.013) | 0.053 (0.003)^#^ | 0.029 (0.008)^#^ | 0.038 (0.012) | <0.01** |

** P<0.01; #: Statistically different from the remaining groups; SEM = Standard error of the mean
